# Comparative Proteomic Analysis of Aminoglycosides Resistant and Susceptible *Mycobacterium tuberculosis* Clinical Isolates for Exploring Potential Drug Targets

**DOI:** 10.1371/journal.pone.0139414

**Published:** 2015-10-05

**Authors:** Divakar Sharma, Bhavnesh Kumar, Manju Lata, Beenu Joshi, Krishnamurthy Venkatesan, Sangeeta Shukla, Deepa Bisht

**Affiliations:** 1 Department of Biochemistry, National JALMA Institute for Leprosy and Other Mycobacterial Diseases, Tajganj, Agra, India; 2 Department of Immunology, National JALMA Institute for Leprosy and Other Mycobacterial Diseases, Tajganj, Agra, India; 3 School of Studies in Zoology, Jiwaji University, Gwalior, India; Tulane University, UNITED STATES

## Abstract

Aminoglycosides, amikacin (AK) and kanamycin (KM) are second line anti-tuberculosis drugs used to treat tuberculosis (TB) and resistance to them affects the treatment. Membrane and membrane associated proteins have an anticipated role in biological processes and pathogenesis and are potential targets for the development of new diagnostics/vaccine/therapeutics. In this study we compared membrane and membrane associated proteins of AK and KM resistant and susceptible *Mycobacterium tuberculosis* isolates by 2DE coupled with MALDI-TOF/TOF-MS and bioinformatic tools. Twelve proteins were found to have increased intensities (PDQuest Advanced Software) in resistant isolates and were identified as ATP synthase subunit alpha (Rv1308), Trigger factor (Rv2462c), Dihydrolipoyl dehydrogenase (Rv0462), Elongation factor Tu (Rv0685), Transcriptional regulator MoxR1(Rv1479), Universal stress protein (Rv2005c), 35kDa hypothetical protein (Rv2744c), Proteasome subunit alpha (Rv2109c), Putative short-chain type dehydrogenase/reductase (Rv0148), Bacterioferritin (Rv1876), Ferritin (Rv3841) and Alpha-crystallin/HspX (Rv2031c). Among these Rv2005c, Rv2744c and Rv0148 are proteins with unknown functions. Docking showed that both drugs bind to the conserved domain (Usp, PspA and SDR domain) of these hypothetical proteins and GPS-PUP predicted potential pupylation sites within them. Increased intensities of these proteins and proteasome subunit alpha might not only be neutralized/modulated the drug molecules but also involved in protein turnover to overcome the AK and KM resistance. Besides that Rv1876, Rv3841 and Rv0685 were found to be associated with iron regulation signifying the role of iron in resistance. Further research is needed to explore how these potential protein targets contribute to resistance of AK and KM.

## Introduction

*Mycobacterium tuberculosis* is the etiological factor of tuberculosis (TB), causes significant morbidity and mortality worldwide. In 2013, WHO reported 8.6 million people developed TB and 1.3 million died from the disease [1]. Increasing spreads of multidrug-resistant tuberculosis (MDR-TB) has worsened the situation and treatment of MDR-TB leads to the use of second line drugs. Emergence of extensively drug resistant tuberculosis (XDR-TB) indicates not only search for new diagnostic markers, drugs, amendment in second line treatment regimens but also to explore the unknown mechanisms of resistance in *M*. *tuberculosis* for developing novel drug targets. Aminoglycosides, AK and KM are important anti-mycobacterial drugs for category-II TB patients. Category II TB patients include those who had failed previous TB treatment, relapsed after treatment, or defaulted during previous treatment. Cumulative mechanisms associated with resistance to aminoglycosides include majorly mutation in ribosomal protein/16S rRNA [2], cell wall impermeability [3], enzymatic inactivation of drugs [4], trapping of drug [5], decreased inner membrane transport and active efflux pumps [6]. Two-third of *M*. *tuberculosis* isolates showed KM and AK resistance due to *rrs* mutation, however remaining 1/3^rd^ do not have these mutations suggesting the involvement of some other mechanism(s) for resistance. Developments in molecular and cellular biology have imposed doubts on the ability of genetic analysis alone to predict any complex phenotypes. As primarily proteins manifest most of the biological processes, information about the actual state of cell can be obtained by analyzing the protein patterns. 2-DE coupled with MALDI-TOF-MS and bioinformatic tools have now been accepted as major analytical tools for detection, identification and characterization of protein species [7–8]. Most of the published proteomic studies concentrate mainly on soluble proteins and there are few comprehensive reports [9–14] on membrane proteins. The identification and characterization of membrane or membrane associated proteins of *M*. *tuberculosis* is important due to their anticipated role in virulence and bacterial-host interactions. Membranes and membrane associated proteins are likely to function as enzymes, receptors, transporters or signal transducers that could be of vital importance to the microbe and hence could qualify as drug targets [15–18]. Comparative proteomic studies addressing whole cell proteins with second line aminoglycosides drug resistance isolates have been reported [8]. However, membrane and membrane associated proteome of aminoglycosides resistant *M*. *tuberculosis* isolates have not been addressed. To address this, we analyzed the membranes and membrane associated proteins of AM and KM resistant *M*. *tuberculosis* by proteomic and bioinformatic approach. Such information could be helpful for the development of newer diagnostics and therapeutic agents for better treatment particularly drug resistance TB.

## Materials and Methods

### *M*. *tuberculosis* isolates and drug susceptibility testing

Five total suseptible (rifampicin, isoniazid, ethambutol, pyrazinamide, streptomycin, kanamycin and amikacin) and five AK & KM resistant (sensitive to first line drugs) *M*. *tuberculosis* isolates were obtained from Mycobacterial Repository Centre of National JALMA Institute for Leprosy and Other Mycobacterial Diseases, Agra, India. Drug susceptibility testing (DST) for all the drugs were performed by LJ proportion [19] and REMA method [20–21]. REMA method uses the oxidation–reduction of colorimetric indicator resazurin for determination of drug resistance and minimal inhibitory concentration (MICs) of antimicrobial agents against *M*. *tuberculosis*. Resazurin, which is blue in its oxidized state, turns pink when reduced by viable cells.

### Membrane and membrane associated protein fraction preparation

Mycobacterial cell lysate was prepared as described by [8 & 22] with slight modifications. Briefly, cells were suspended in sonication buffer with 1% v/v Triton X–100 and then broken by intermittent sonication at 4°C for 20 min. Homogenate was centrifuged at 12,000 g for 20 min at 4°C. Resulting supernatants were ultracentrifuged at 150,000 x *g* for 90 min. and the pellet (cell membrane) was collected, washed and dissolved in 2D rehydration buffer. Protein concentrations were estimated by Bradford method [23] using BSA as standard. Protein extractions were performed for three times in biological and technical replicas.

### 2DE, In gel digestion & MS

IEF & SDS-PAGE were carried out using the published protocol of “in gel rehydration” with slight modifications [8 & 24]. Gel images were analyzed using PDQuest Advanced software version 8.0.0 (BIORAD, Hercules, CA, USA). Protein spots which showed increased intensities with more than 1.5 fold were selected for identification. Equal amount of proteins were loaded in all gels and experiments were repeated in biological and technical replicates at least three times. In-gel digestion of proteins and MALDI-TOF/MS was carried out using published protocol [8 & 25]. Mass spectra of digested proteins were acquired using Autoflex II TOF/TOF 50 (Bruker Daltonik GmbH, Leipzig, Germany).

### Validation by MS/MS analysis

Matched precursor peptide ions of identified proteins were selected for subsequent fragmentation using PSD for MS/ MS. Lift_ATT.lift method was open in flex control software; parent peak mass spectrum was acquired by hitting laser for 400–550 shots followed by acquisition of fragments of selected precursor ion for the same no. of shots. Both parent and fragment spectrums were pooled to generate MS/MS spectrum of a particular peptide. MS/MS spectrum was submitted to database using MASCOT wizard described in MS protocol [8]. The same parameters were used for MS/MS search in addition with fragment mass tolerance from 0.2 to 1.0 Da.

### Bioinformatic analysis

Protein sequences of selected proteins were retrieved from Tuberculist server http://tuberculist.epfl.ch/ and their probable functions were predicted using published protocol of BLASTp, InterProScan, KEGG, docking and GPS-PUP [26–31].

## Results

**Fig 1:**
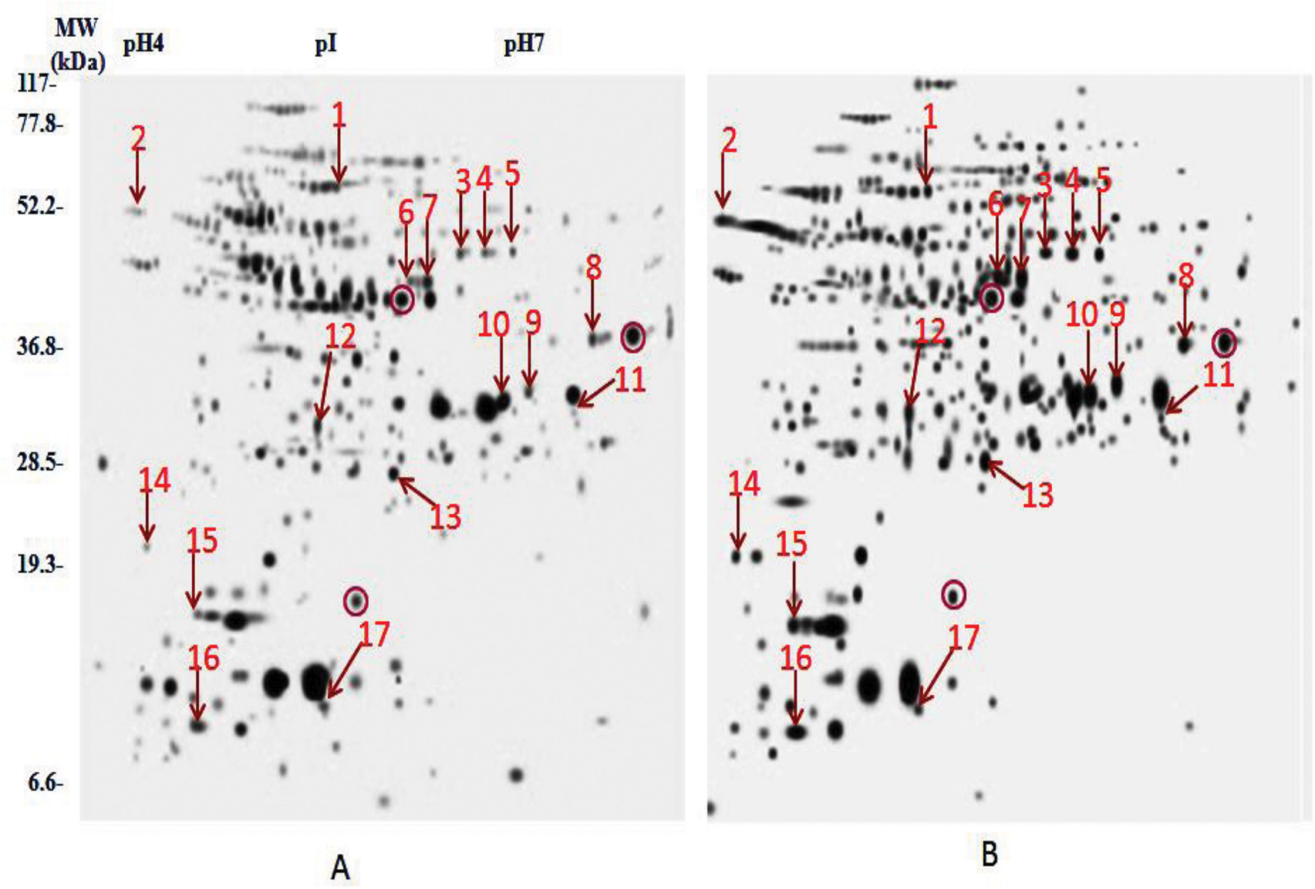
Composite images of 2DE profile *M*. *tuberculosis isolates* (a) Total susceptible (b) AM and KM resistant (Encircled spots are taken as internal control).

**Fig 2:**
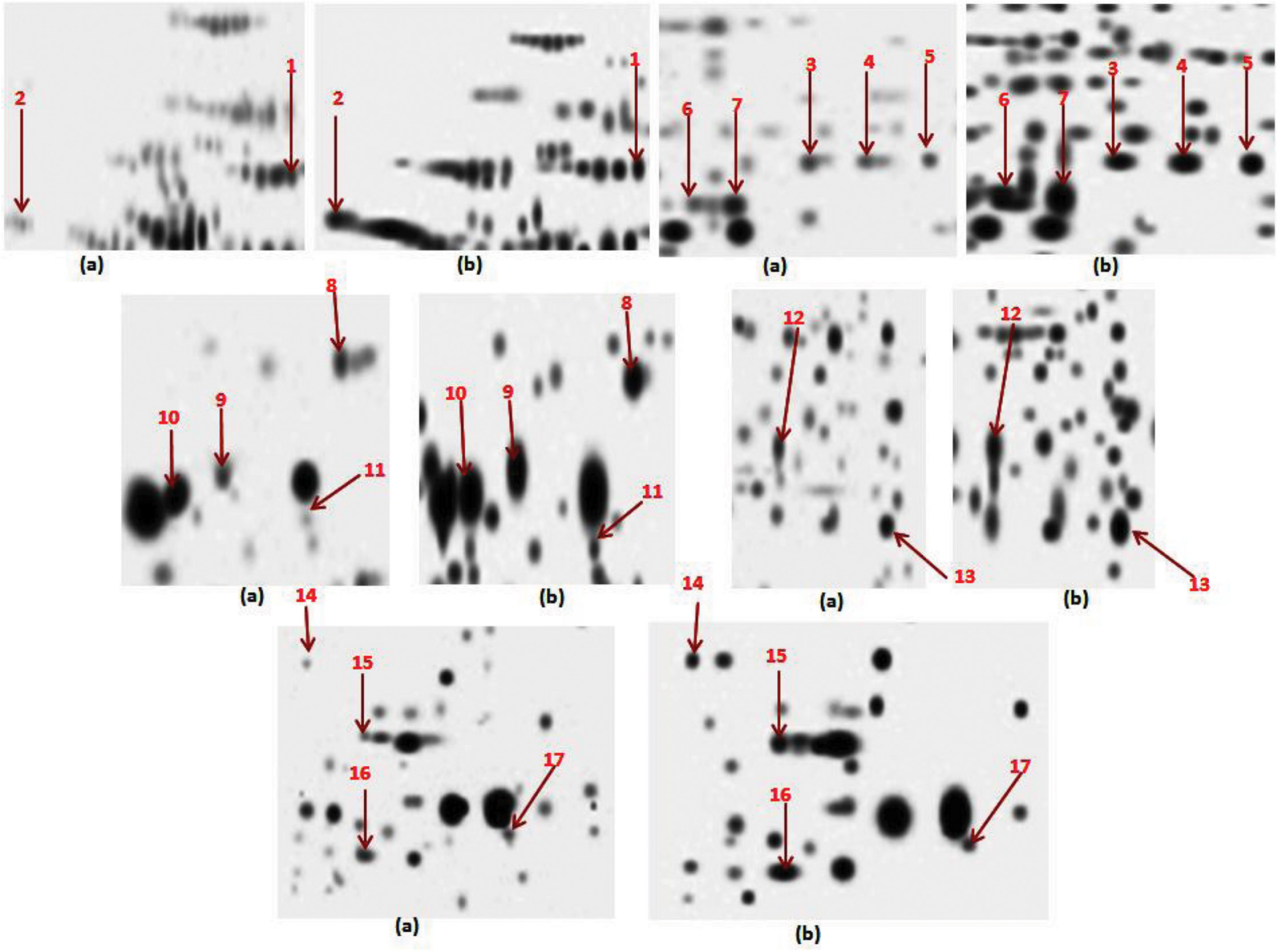
Magnified regions of 2D gels showing proteins of increased intensity (a) Sensitive (b) Resistant.

The main aim of the study was to compare the membranes and membrane associated proteins profiles of AM and KM resistant (lacks *rrs* mutation) with total sensitive isolates. *rrs* gene encoding 16S rRNA, have been associated with amikacin and kanamycin resistance. Results of DST by REMA methods are represented in Table 1. 2DE profile run in triplicates for all isolates was employed to compare the protein profiles and composite images are shown in Fig 1. Comparison of 2D gels by PDQuest Advanced software revealed seventeen protein spots (identified as twelve protein with its species) with consistently increased intensities in resistant as compared to sensitive isolates (cut limit ≥ 1.5 fold change in spot intensity). Student t-test was used for the statistical analysis by PDQuest Advanced software. The system picks up the spots with differential intensity of significant levels built in the system. To rule out the chance of any artifact, proteins showing equal intensity were considered as internal control (encircled in Fig 1). Protein spots encircled in Fig 1 were taken as internal controls to monitor the equal loading on the gels. Magnified regions of these protein spots are shown in Fig 2. Proteins spots of increased intensities were identified by MALDI-TOF-MS (Table 2) and their identity were further revalidated by MS/MS (Table 3) taking at least three peptides to be matched. Detailed information of MS and MS/MS of all the proteins were shown in supporting files (S1 Text and S2 Text). The identified proteins were ATP synthase subunit alpha (Rv1308), Trigger factor (Rv2462c), Dihydrolipoyl dehydrogenase (Rv0462), Elongation factor Tu (Rv0685), Transcriptional regulator MoxR1(Rv1479), Universal stress protein (Rv2005c), 35kDa hypothetical protein (Rv2744c), Proteasome subunit alpha (Rv2109c), Putative short-chain type dehydrogenase/reductase (Rv0148), Bacterioferritin (Rv1876), Ferritin (Rv3841) and Alpha-crystallin/HspX (Rv2031c). Out of twelve, Rv1308, Rv0462, Rv2109c, Rv0148, Rv1876 and Rv3841 belonged to intermediary metabolism and respiration, Rv2005c and Rv2031c to virulence/detoxification/adaptation, Rv2462c to cell wall and cell processes, Rv2744c to conserved hypothetical, Rv1479 to regulatory proteins and Rv0685 to information pathways categories. The level of difference in protein spot intensity has been represented as densitometric ratio in Table 2. These proteins were also reported in membrane fraction of *M*. *tuberculosis* complex by various authors [9–14].

**Table 1:** Drug susceptibility profile of *M*. *tuberculosis* isolates included in this study.

S. No.	Isolates Code	Drug susceptibility profile by Proportion method						MIC by REMA method		
		SM	RIF	INH	EMB	PZA	KM	SM μg/ml	AK μg/ml	KM μg/ml
**1**	H_37_Rv	S	S	S	S	S	S	≤0.2	≤0.025	0.05
**2**	S 1	S	S	S	S	S	S	2.0	0.1	0.1
**3**	S 2	S	S	S	S	S	S	0.5	0.2	0.2
**4**	S 3	S	S	S	S	S	S	≤0.2	≤0.025	0.05
**5**	S 4	S	S	S	S	S	S	2.0	0.2	0.1
**6**	S 5	**S**	S	S	S	S	S	1.0	0.1	0.2
**7**	R 1	**S**	S	S	S	S	**R**	0.5	12	16
**8**	R 2	**S**	S	S	S	S	**R**	≤0.2	16	32
**9**	R 3	**S**	S	S	S	S	**R**	1.0	16	16
**10**	R 4	**S**	S	S	S	S	**R**	1.0	32	12
**11**	R 5	**S**	S	S	S	S	**R**	≤0.2	12	32

S: sensitive; R: resistant; Rifampicin (RIF), Isoniazid (INH), Ethambutol (EMB), Streptomycin (SM), Pyrazinamide (PZA), Amikacin (AK), Kanamycin (KM)

**Table 2:** Details of proteins identified by Mass Spectrometry.

Spot No.	Accession Number	Protein identified	MASCOT Score	Nominal Mass (Da)	pI	Sequence Coverage %	ORF No.	Densitometric ratio of protein intensity between sensitive and resistant isolates	Functional category *
**D1**	P63673 (ATPA_MYCTU)	ATP synthase subunit alpha	172	59252	5.03	35%	Rv1308	1: 1.54	1
**D 2**	O53189 (TIG_MYCTU)	Trigger factor	55	50586	4.43	21%	Rv2462c	1: 1.80	2
**D 3**	P66004 (DLDH_MYCTU)	Dihydrolipoyl dehydrogenase	125	49208	5.53	35%	Rv0462	1: 1.62	1
**D 4**	P66004 (DLDH_MYCTU)	Dihydrolipoyl dehydrogenase	116	49208	5.53	35%	Rv0462	1: 1.53	1
**D 5**	P66004 (DLDH_MYCTU)	Dihydrolipoyl dehydrogenase	52	49208	5.53	13%	Rv0462	1: 1.60	1
**D 6**	P0A558 (EFTU_MYCTU)	Elongation factor Tu	193	43566	5.28	60%	Rv0685	1: 1.58	3
**D 7**	P0A558 (EFTU_MYCTU)	Elongation factor Tu	143	43566	5.28	50%	Rv0685	1: 1.73	3
**D 8**	Q79FN7 (Q79FN7_MYCTU)	Transcriptional regulator MoxR1	116	40738	5.96	32%	Rv1479	1: 1.52	4
**D 9**	P64921 (Y2005_MYCTU)	Universal stress protein	61	30966	5.53	23%	Rv2005c	1: 1.99	5
**D 10**	P0C5C4 (35KD_MYCTU)	35kDa protein	124	29240	5.71	33%	Rv2744c	1: 2.00	6,2
**D11**	P0C5C4 (35KD_MYCTU)	35kDa protein	79	29240	5.71	61%	Rv2744c	1: 1.69	6,2
**D 12**	O33244 (PSA_MYCTU)	Proteasome subunit alpha	69	26865	5.41	27%	Rv2109c	1: 2.09	1
**D 13**	P96825 (Y0148_MYCTU)	Putative short-chain type dehydrogenase/reductase	181	29760	5.26	59%	Rv0148	1: 1.91	1
**D14**	P63697 (BFR_MYCTU)	Bacterioferritin	54	18239	4.50	27%	Rv1876	1: 2.70	1
**D 15**	P96237 (BFRB_MYCTU)	Ferritin	114	20429	4.73	38%	Rv3841	1: 1.83	1
**D 16**	P0A5B7 (ACR_MYCTU)	Alpha-crystallin	148	16217	5.00	78%	Rv2031c	1: 1.64	5
**D 17**	P0A5B7 (ACR_MYCTU)	Alpha-crystallin	93	16217	5.00	54%	Rv2031c	1: 1.98	5

*Note: 1- intermediary metabolism and respiration, 2- cell wall and cell processes, 3- information pathways, 4- regulatory proteins, 5- virulence, detoxification, adaptation, 6- conserved hypothetical’s

**Table 3:** MS/MS analysis of identified proteins.

Spot No.	Peak Mass (Da)	Protein Identified	Nominal Mass	Mascot Score	pI	Sequence of peptides	ORF No.
**D1**	894.4477	ATP synthase subunit alpha	59252	25	5.03	LDLSQYR	Rv1308
	1264.7051	ATP synthase subunit alpha	59252	18	5.03	VVNPLGQPIDGR	Rv1308
	1289.6714	ATP synthase subunit alpha	59252	21	5.03	ASEEEILTEIR	Rv1308
	1297.7342	ATP synthase subunit alpha	59252	19	5.03	QGVKEPLQTGIK	Rv1308
	1313.7349	ATP synthase subunit alpha	59252	60	5.03	HVLIIFDDLTK	Rv1308
	1319.7631	ATP synthase subunit alpha	59252	30	5.03	ALELQAPSVVHR	Rv1308
	1553.7943	ATP synthase subunit alpha	59252	48	5.03	EAYPGDVFYLHSR	Rv1308
	1602.9144	ATP synthase subunit alpha	59252	65	5.03	TGEVLSVPVGDGFLGR	Rv1308
	1747.9579	ATP synthase subunit alpha	59252	52	5.03	ASEEEILTEIRDSQK	Rv1308
	1886.0888	ATP synthase subunit alpha	59252	98	5.03	LSDDLGGGSLTGLPIIETK	Rv1308
	2612.4725	ATP synthase subunit alpha	59252	51	5.03	GFAATGGGSVVPDEHVEALDEDKLAK	Rv1308
**D2**	1291.7448	Trigger factor	50586	25	4.43	NQLPTMFADVR	Rv2462c
	1378.8073	Trigger factor	50586	32	4.43	FNELLVEQGSSR	Rv2462c
	1671.9877	Trigger factor	50586	81	4.43	EAMLDQIVNDALPSR	Rv2462c
	1801.1078	Trigger factor	50586	94	4.43	LIAGLDDAVVGLSADESR	Rv2462c
	1903.1486	Trigger factor	50586	109	4.43	INVEVPFAELEPDFQR	Rv2462c
	2158.3327	Trigger factor	50586	29	4.43	VRINVEVPFAELEPDFQR	Rv2462c
**D3**	1621.8785	Dihydrolipoyl dehydrogenase	49208	118	5.53	NYGVDVTIVEFLPR	Rv0462
	1890.9713	Dihydrolipoyl dehydrogenase	49208	63	5.53	VLQAIGFAPNVEGYGLDK	Rv0462
	1909.9795	Dihydrolipoyl dehydrogenase	49208	48	5.53	SIIIAGAGAIGMEFGYVLK	Rv0462
	1980.9351	Dihydrolipoyl dehydrogenase	49208	50	5.53	AFGISGEVTFDYGIAYDR	Rv0462
	2015.0726	Dihydrolipoyl dehydrogenase	49208	13	5.53	THYDVVVLGAGPGGYVAAIR	Rv0462
	2276.1980	Dihydrolipoyl dehydrogenase	49208	100	5.53	LVPGTSLSANVVTYEEQILSR	Rv0462
	2688.4487	Dihydrolipoyl dehydrogenase	49208	25	5.53	LGVTILTATKVESIADGGSQVTVTVTK.D	Rv0462
	2774.4226	Dihydrolipoyl dehydrogenase	49208	111	5.53	HGELLGGHLVGHDVAELLPELTLAQR	Rv0462
**D4**	1161.6462	Dihydrolipoyl dehydrogenase	49208	21	5.53	WDLTASELAR	Rv0462
	1171.6699	Dihydrolipoyl dehydrogenase	49208	39	5.53	NAELVHIFTK	Rv0462
	1621.9626	Dihydrolipoyl dehydrogenase	49208	51	5.53	NYGVDVTIVEFLPR	Rv0462
	1891.1342	Dihydrolipoyl dehydrogenase	49208	85	5.53	VLQAIGFAPNVEGYGLDK	Rv0462
	1981.1184	Dihydrolipoyl dehydrogenase	49208	14	5.53	AFGISGEVTFDYGIAYDR	Rv0462
	2015.2435	Dihydrolipoyl dehydrogenase	49208	15	5.53	THYDVVVLGAGPGGYVAAIR	Rv0462
**D5**	895.5120	Dihydrolipoyl dehydrogenase	49208	19	5.53	FPFTANAK	Rv0462
	1161.7045	Dihydrolipoyl dehydrogenase	49208	28	5.53	WDLTASELAR	Rv0462
	1170.6847	Dihydrolipoyl dehydrogenase	49208	20	5.53	AHGVGDPSGFVK	Rv0462
	1171.7299	Dihydrolipoyl dehydrogenase	49208	33	5.53	NAELVHIFTK	Rv0462
	1397.9056	Dihydrolipoyl dehydrogenase	49208	19	5.53	AAQLGLSTAIVEPK	Rv0462
**D6**	1404.5958	Elongation factor Tu	43566	24	5.28	AFDQIDNAPEER	Rv0685
	1413.7572	Elongation factor Tu	43566	67	5.28	QVGVPYILVALNK	Rv0685
	1555.7710	Elongation factor Tu	43566	34	5.28	VLHDKFPDLNETK	Rv0685
	1701.8430	Elongation factor Tu	43566	28	5.28	GITINIAHVEYQTDK	Rv0685
	1801.8729	Elongation factor Tu	43566	76	5.28	.ELLAAQEFDEDAPVVR	Rv0685
	2074.9777	Elongation factor Tu	43566	53	5.28	ADAVDDEELLELVEMEVR	Rv0685
	2195.1034	Elongation factor Tu	43566	31	5.28	ETDKPFLMPVEDVFTITGR	Rv0685
	2356.1634	Elongation factor Tu	43566	34	5.28	WVASVEELMNAVDESIPDPVR	Rv0685
**D7**	1404.6006	Elongation factor Tu	43566	28	5.28	AFDQIDNAPEER	Rv0685
	1413.7786	Elongation factor Tu	43566	70	5.28	QVGVPYILVALNK	Rv0685
	1681.8413	Elongation factor Tu	43566	110	5.28	LLDQGQAGDNVGLLLR	Rv0685
	1801.8014	Elongation factor Tu	43566	57	5.28	ELLAAQEFDEDAPVVR	Rv0685
	2033.8394	Elongation factor Tu	43566	15	5.28	HTPFFNNYRPQFYFR	Rv0685
	2074.8391	Elongation factor Tu	43566	48	5.28	ADAVDDEELLELVEMEVR	Rv0685
	2194.9570	Elongation factor Tu	43566	52	5.28	ETDKPFLMPVEDVFTITGR	Rv0685
	2355.9810	Elongation factor Tu	43566	40	5.28	WVASVEELMNAVDESIPDPVR	Rv0685
**D8**	1785.8129	Transcriptional regulator MoxR1	40738	27	5.96	IQFTPDLVPTDIIGTR	Rv1479
	1982.8948	Transcriptional regulator MoxR1	40738	29	5.96	DYVIPQDVIEVIPDVLR	Rv1479
	2196.0884	Transcriptional regulator MoxR1	40738	43	5.96	GRDYVIPQDVIEVIPDVLR	Rv1479
	2244.1285	Transcriptional regulator MoxR1	40738	94	5.96	LVLTYDALADEISPEIVINR	Rv1479
	2308.0928	Transcriptional regulator MoxR1	40738	37	5.96	LQEIAANNFVHHALVDYVVR	Rv1479
	2491.0784	Transcriptional regulator MoxR1	40738	21	5.96	EEFDTELGPVVANFLLADEINR	Rv1479
	2832.2880	Transcriptional regulator MoxR1	40738	65	5.96	QGREEFDTELGPVVANFLLADEINR	Rv1479
**D9**	859.4570	Universal stress protein	30966	21	5.53	LAGWQER	Rv2005c
	932.4974	Universal stress protein	30966	56	5.53	YPDVPVSR	Rv2005c
	1330.8178	Universal stress protein	30966	11	5.53	GLLGSVSSSLVRR	Rv2005c
	1367.8154	Universal stress protein	30966	44	5.53	SASAQLVVVGSHGR	Rv2005c
	1737.0408	Universal stress protein	30966	53	5.53	LAGWQERYPDVPVSR	Rv2005c
	1924.1982	Universal stress protein	30966	44	5.53	GGLTGMLLGSVSNAVLHAAR	Rv2005c
**D10**	1029.5576	35 kDa protein	29240	20	5.71	LLSQLEQAK	Rv2744c
	1412.7607	35 kDa protein	29240	72	5.71	VQIQQAIEEAQR	Rv2744c
	1424.7377	35 kDa protein	29240	13	5.71	TLHDQALSAAAQAK	Rv2744c
	1525.8869	35 kDa protein	29240	56	5.71	QLADIEKLQVNVR	Rv2744c
	1615.8335	35 kDa protein	29240	10	5.71	QALTLADQATAAGDAAK	Rv2744c
	1822.9247	35 kDa protein	29240	132	5.71	YANAIGSAELAESSVQGR	Rv2744c
	2783.3882	35 kDa protein	29240	84	5.71	ATEYNNAAEAFAAQLVTAEQSVEDLK	Rv2744c
**D11**	1412.8493	35 kDa protein	29240	50	5.71	VQIQQAIEEAQR	Rv2744c
	1525.9723	35 kDa protein	29240	49	5.71	QLADIEKLQVNVR	Rv2744c
	1823.0114	35 kDa protein	29240	21	5.71	YANAIGSAELAESSVQGR	Rv2744c
	1864.0864	35 kDa protein	29240	136	5.71	THQALTQQAAQVIGNQR	Rv2744c
**D12**	1054.5026	Proteasome subunit alpha	26865	25	5.41	FNEFDNLR	Rv2109c
	2020.1235	Proteasome subunit alpha	26865	74	5.41	SVVALAYAGGVLFVAENPSR	Rv2109c
	2219.2512	Proteasome subunit alpha	26865	24	5.41	AKSVVALAYAGGVLFVAENPSR	Rv2109c
	2924.3203	Proteasome subunit alpha	26865	29	5.41	AGSADTSGGDQPTLGVASLEVAVLDANRPR	Rv2109c
**D13**	884.4423	Putative short-chain type dehydrogenase/reductase	29760	13	5.26	AAWPHFR	Rv0148
	1190.5693	Putative short-chain type dehydrogenase/reductase	29760	27	5.26	WAEITDLSGAK	Rv0148
	1313.6913	Putative short-chain type dehydrogenase/reductase	29760	23	5.26	VHLYGGYHVLR	Rv0148
	1339.6028	Putative short-chain type dehydrogenase/reductase	29760	53	5.26	MSFENWDAVLK	Rv0148
	1581.9283	Putative short-chain type dehydrogenase/reductase	29760	90	5.26	LGLVGLINTLALEGAK	Rv0148
	1748.8280	Putative short-chain type dehydrogenase/reductase	29760	14	5.26	DGTGAGSAMADEVVAEIR	Rv0148
	1921.9303	Putative short-chain type dehydrogenase/reductase	29760	36	5.26	AVANYDSVATEDGAANIIK	Rv0148
	2162.1125	Putative short-chain type dehydrogenase/reductase	29760	102	5.26	EYALTLAGEGASVVVNDLGGAR	Rv0148
	2388.1836	Putative short-chain type dehydrogenase/reductase	29760	58	5.26	VALFGNDGANFDKPPSVQDVAAR	Rv0148
**D14**	1414.6818	Bacterioferritin	18239	79	4.50	ILLLDGLPNYQR	Rv1876
	1776.6893	Bacterioferritin	18239	55	4.50	MQDNWGFTELAAHTR	Rv1876
	1924.8015	Bacterioferritin	18239	45	4.50	EQFEADLAIEYDVLNR	Rv1876
**D15**	932.4086	Ferritin	20429	31	4.73	NQFDRPR	Rv3841
	1084.5530	Ferritin	20429	54	4.73	VEIPGVDTVR	Rv3841
	1228.6306	Ferritin	20429	27	4.73	EALALALDQER	Rv3841
	1265.5885	Ferritin	20429	19	4.73	HFYSQAVEER	Rv3841
	1550.8525	Ferritin	20429	105	4.73	AGANLFELENFVAR	Rv3841
	1632.8621	Ferritin	20429	15	4.73	EVDVAPAASGAPHAAGGR	Rv3841
**D16**	1095.5822	Alpha-crystallin	16217	36	5.00	TEQKDFDGR	Rv2031c
	1162.6453	Alpha-crystallin	16217	66	5.00	SEFAYGSFVR	Rv2031c
	1715.0573	Alpha-crystallin	16217	42	5.00	GILTVSVAVSEGKPTEK	Rv2031c
	1752.8950	Alpha-crystallin	16217	96	5.00	DFDGRSEFAYGSFVR	Rv2031c
	1869.0448	Alpha-crystallin	16217	19	5.00	AELPGVDPDKDVDIMVR	Rv2031c
	2037.1053	Alpha-crystallin	16217	29	5.00	TVSLPVGADEDDIKATYDK	Rv2031c
	2950.6204	Alpha-crystallin	16217	13	5.00	SLFPEFSELFAAFPSFAGLRPTFDTR	Rv2031c
**D17**	1162.4795	Alpha-crystallin	16217	65	5.00	SEFAYGSFVR	Rv2031c
	1458.6522	Alpha-crystallin	16217	17	5.00	TVSLPVGADEDDIK	Rv2031c
	1714.8305	Alpha-crystallin	16217	43	5.00	GILTVSVAVSEGKPTEK	Rv2031c
	1752.6882	Alpha-crystallin	16217	32	5.00	DFDGRSEFAYGSFVR	Rv2031c
	1868.8202	Alpha-crystallin	16217	53	5.00	AELPGVDPDKDVDIMVR	Rv2031c
	2036.8814	Alpha-crystallin	16217	56	5.00	TVSLPVGADEDDIKATYDK	Rv2031c
	2293.0996	Alpha-crystallin	16217	17	5.00	ATYDKGILTVSVAVSEGKPTEK	Rv2031c

### BLAST and InterProScan analysis

BLASTP analysis was performed for proteins of unknown function. Rv0148 was found to be highly conserved in mycobacterial and bacterial species as putative short chain dehydrogenase/reductase protein. InterProScan analysis of Rv0148 showed motifs (PF00106) from residues 8–183 which provides a signature for short chain dehydrogenase. Rv2005c was found to be highly conserved in mycobacterial and bacterial species as universal stress protein, in some mycobacterial species it appeared as hypothetical protein with unknown function. InterProScan analysis of Rv2005c showed the presence of two signature motifs of Usp domain with amino acid residues from10-148 and 162–293 (PF00582) and three signatures motifs of universal stress protein with amino acid residues from 159–177, 253–265 and 271–293 (PRINTS: PR01438). Rv2744c was found to be conserved alanine rich hypothetical protein, exhibited significant homology with hypothetical and phase shock protein A (pspA) of all *M*. *tuberculosis complex* and NTMs. InterProScan analysis of Rv2744c showed the presence of PspA domain with amino acid residues from 3–242 (PF04012).

### Multiple Sequence Alignment

Multiple sequence alignment of mtu (*M*. *tuberculosis*) proteins was performed for the set of five organism’s mbo (*M*. *bovis*), maf (*M*. *africanum*), mav (*M*. *avium*), mle (*M*. *leprae*) and hsa (*Homo sapiens*) {Table 4}. Results showed that hypothetical proteins (Rv0148, Rv2005c and Rv2744c) exhibited 100% homology (except Rv2744c- 99.60% and 99.30% homology) to their corresponding proteins in *M*. *bovis* and *M*. *africanum* which are members of tuberculosis complex. In *M*. *avium*, > 87% homology has been seen, except Rv2005c (64.40%). Less than 48% of homology has been seen with *M*. *leprae* and *Homo sapiens* to their corresponding proteins as well as with other proteins.

**Table 4:** Multiple sequence alignment of the hypothetical proteins with defined set of organisms.

ORF Number	Mbo (*M*. *bovis*)	Maf (*M*. *africanum*)	Mav (*M*. *avium*)	Mle (*M*. *leprae*)	Has (*Homo*. *sapiens*)
**Rv0148**	100%	100%	87.30%	23.42%	47.50%
**Rv2005c**	100%	100%	64.40%	28.10%	21.70%
**Rv2744c**	99.6%	99.30%	88.00%	26.60%	24.60%

### 3D modeling and docking

**Fig 3:**
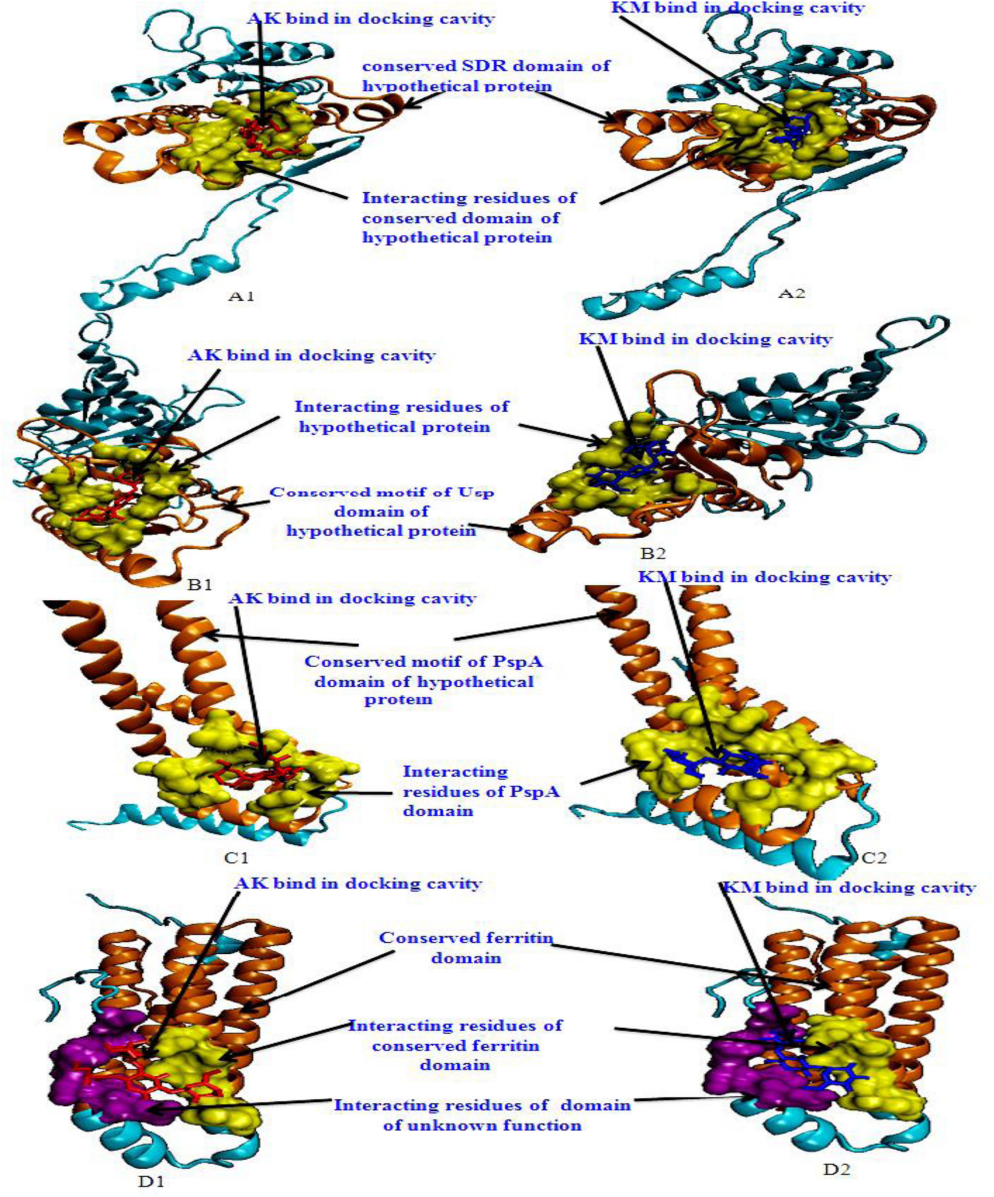
3D model of hypothetical proteins & ferritin showing docking with AK & KM: A1 and A2 shows molecular docking of Rv0148 with AM (red) & KM (blue) respectively, orange color shows SDR domain, yellow color shows interacting residues of SDR domain. B1 and B2 shows molecular docking of Rv2005c with AM (red) & KM (blue) respectively, orange color shows Usp domain, yellow color shows interacting residues of Usp domain. C1 and C2 shows docking of Rv2744c with AM (red) & KM (blue) respectively, orange color shows PspA domain of hypothetical protein, yellow color shows interacting residues of PspA domain. D1 and D2 shows docking of Rv3841 with AM (red) & KM (blue) respectively, orange color shows conserved ferritin domain of protein, yellow color shows interacting residues of conserved ferritin domain, purple color shows interacting residues of unknown domain.

Molecular docking analysis of selected 3D models (showing less than 2% discrepancy from Ramachandran plot) of hypothetical proteins was performed to detect their binding with AK and KM. Parameters used for selection of 3D models and molecular docking are represented in Table 5. Docking of Rv0148 and Rv2005c (Fig 3) showed the interaction of both drugs into the central cavity of conserved motif of SDR domain and Usp domain of hypothetical proteins respectively. Interacting residues were almost common for both drugs, which suggests similar binding site for both. Docking with Rv2744c show that both drugs interact at the similar interacting residue of conserved PspA domain of hypothetical protein. With Rv3841 both drugs interacted with amino acids of conserved ferritin domain as well as domain of unknown function.

**Table 5:** 3D modeling and docking parameters used for bioinformatic analysis.

ORF No.	TM-score	RMSD value (Å)	Drug	Global Energy	Attractive Vander wall forces	Repulsive Vander wall forces	ACE	Interacting amino acids	Remarks
**Rv0148**	0.81±0.09	4.6±3.0Å	AK	-29.30	-20.55	19.86	-10.46	18,99,123,149,150,152,153,158,161,164,168,194–196,198,200 & 201	AK binds properly within the central cavity of conserved SDR domain
**Rv0148**	0.81±0.09	4.6±3.0Å	KM	-45.45	-22.03	10.90	-14.08	99,103,123,149,150–152,164,168,194–196,198,200 & 201	KM also binds within the central cavity of conserved SDR domain
**Rv2005c**	0.95±0.05	2.8±2.0Å	AK	-32.66	-20.26	8.27	-06.23	15,16,42,43,64,67,71,100,101,120,121 & 130–132	AK binds in central cavity of conserved motif of Usp domain
**Rv2005c**	0.95±0.05	2.8±2.0Å	KM	-31.64	-18.15	3.76	-05.29	14,15,16,42,43,44,64,67,71,100,120,121 & 122	KM also binds in central cavity of conserved motif of Usp domain of hypothetical protein
**Rv2744c**	0.30±0.10	15.3±3.4Å	AK	-22.56	-22.44	7.00	00.59	19,20,22,25,29,36,38,39,40,41 & 44	AK interact to conserved motif of PspA domain
**Rv2744c**	0.30±0.10	15.3±3.4Å	KM	-25.82	-16.56	2.90	-03.51	19,20,22,25,29,36,38,39,40,41,44 & 230	KM also interact to conserved motif of PspA domain of hypothetical protein
**Rv3841**	0.92±0.06	2.3±1.7Å	AK	-26.34	-16.62	4.97	-7.23	134,137,138,141,144,163, 164,165,166,167,168,169& 170	AK binds to close vicinity of conserved ferritin domain & domain of unknown function
**Rv3841**	0.92±0.06	2.3±1.7Å	KM	-34.66	-19.57	7.05	-9.35	134,135,137,138,141,144,163,164,165,166,167,168,169 &170	KM also binds to close vicinity of conserved ferritin domain & domain of unknown function

### Prediction of pupylation sites

By utilizing the default threshold (medium), GPS-PUP predicted six pupylation sites at position K7, K71, K94, K120, K134, and K135 in Rv2744c. Rv2005c and Rv0148 showed two pupylation sites at position K80, K248 and K280, K285 respectively (Table 6).

**Table 6:** Predicted / identified pupylation sites within identified proteins.

ORF No.	Position of lysine residue undergoes pupylation	Peptide	Score	Cut-off
Rv2005c	80	ANAVKLA**K**EAVGADR	3.488	2.452
	248	VCDRPAR**K**LVQKSAS	3.858	2.452
Rv0148	280	ITDLSGA**K**IAGFKL*	3.11	2.452
	285	GAKIAGF**K**L******	2.748	2.452
Rv2744c	7	*MANPFV**K**AWKYLMA	3.15	2.452
	71	RQLADIE**K**LQVNVRQ	3.315	2.452
	94	TAAGDAA**K**ATEYNNA	2.787	2.452
	120	EQSVEDL**K**TLHDQAL	3.063	2.452
	134	LSAAAQA**K**KAVERNA	2.835	2.452
	135	SAAAQAK**K**AVERNAM	2.496	2.452

## Discussion

In this study we used a proteomic approach to compare membrane and membrane associated proteins of AK and KM resistant and susceptible isolates by 2DE, MALDI-TOF/MS and bioinformatic tools. Resistant isolates were also sequenced and analyzed for known rrs mutations. These isolates did not exhibit mutations at the reported sites. Proteins with increased intensities in the resistant isolates were identified, which might be used as diagnostic markers or drug targets for therapeutics. 2DE/MS has an advantage over the traditional methods (SDS-PAGE, chromatography and sequencing) as not only the identification of a large number of unknown proteins but also protein species separation. Several reports for identification of diagnostics and drug targets employing proteomic approaches exist [32–33]. However, to the best of our knowledge, no such membrane proteome analysis with AK & KM resistant *M*. *tuberculosis* isolates has been reported.

Our study revealed twelve proteins with increased intensity in AM and KM resistant as compared to total susceptible isolates. Five spots matched with already identified protein species and therefore total seventeen protein spots were found to be upregulated. Out of twelve, Rv1308, Rv0462, Rv2109c, Rv0148, Rv1876 and Rv3841 belonged to intermediary metabolism and respiration, Rv2005c and Rv2031c to virulence/detoxification/adaptation, Rv2462c to cell wall and cell processes, Rv2744c to conserved hypothetical, Rv1479 to regulatory proteins and Rv0685 to information pathways categories. These proteins were membrane associated [9–14] but were not purely membrane proteins having transmembrane helix. This might be due to the selection of consistently increased intensities of spots in resistant as compared to sensitive isolates (cut limit ≥ 1.5 fold changes in spot intensity).This suggests that membranes with trans membrane helix do not show consistently increased intensities up to 1.5 fold.

Rv1308 (ATP synthase subunit alpha) is a regulatory subunit that produces ATP in the presence of proton gradient across the membrane. Mycobacteria reside in specialized niches and may require adaptations in the energy metabolism. It has been reported that it not only stipulates in replicating mycobacteria, but also in the dormant state [34–36]. Rv0462 (Dihydrolipoyl dehydrogenase/Lpd) is involved in energy metabolism and antioxidant defense. It is the third enzyme of *M*. *tuberculosis*’s pyruvate dehydrogenase complex and first enzyme of peroxynitrite reductase/peroxidase, which helps *M*. *tuberculosis* to resist host reactive nitrogen intermediates. Without Lpd, *M*. *tuberculosis* cannot metabolize branched-chain amino acids and potentially toxic branched-chain intermediates accumulate [37]. Heo et al [38] reported that this protein induces dendritic cells maturation, Th1-mediated responses and may contribute to vaccine development against *M*. *tuberculosis* infection. Rv2109c (Proteasome subunit alpha) is involved in protein degradation and required for the virulence of *M*. *tuberculosis*. It not only degrades proteins that are toxic due to oxidative or nitrosative damage but also allows other proteins to participate in NO detoxification or repair of macromolecules [39]. Fortune et al [40] reported its involvement in regulating the synthesis of secreted or surface proteins that alter host immunity and thus favor persistence. Discovery of pupylome in *M*. *tuberculosis* [41] and other conserved proteasomal components like proteasome b/a subunits, recognition ATPase, pupylase, and depupylome revealed the protein regulation and turnover through proteasome. Identity of these proteins began to explain why defects in protein degradation attenuate virulence *in vivo* [42–43]. Rv0148 has been identified as probable short-chain dehydrogenases/reductases and possess two binding domains- NAD and substrate. *M*. *tuberculosis* acquires Rv0148 gene via horizontal gene transfer from eukaryotics. As the gene has been retained in the genome through selective advantage, it might play a key role in pathogenesis and immunomodulation [44].

Rv1876 (bacterioferritin) and Rv3841 (ferritin), unique for iron homeostasis and are increased intensities under iron-rich and decreased under iron deprived conditions [45]. Very little is known about the protein-protein interactions that carry iron for storage or promote the mobilization of stored iron from ferritin like molecules. Iron assimilation and utilization in *M*. *tuberculosis* plays a crucial role in growth, virulence and latency. Function of these may not be just limited to iron uptake; they may be contributing to other metabolic activities, the mechanism of which is still unclear. Pandey and Rodriguez [46] suggested that ferritin (bfrB) is mandatory to maintain iron homeostasis in *M*. *tuberculosis* and ferritin lacking bacilli are more susceptible to killing by antibiotics. Our results showed increased intensities of both ferritin and bacterioferritin in membrane fraction. Earlier we reported that only bacterioferritin intensity increased in whole cell lysate and suggested its role in imparting resistance to AK and KM [8]. It is assumed that the heme group present might seize its site of action by providing abnormal site for binding or modulating the protein to block its binding site which needs to be further explored. Although the pathways and enzymes involved in iron metabolism in *M*. *tuberculosis* are well established, still our information on iron dependent post-transcriptional, translational regulations, outer membrane iron transporters, trafficking and partitioning of siderophores/Fe-loaded apoproteins in mycobacteria is inadequate. Consequently, it can be a promising antimycobacterial target.

Rv2031c (Alpha-crystallin/HspX), a heat shock protein and Rv2005c (universal stress protein) are not only involved in cell protection to diverse stimuli like stress, dormancy, heat, drug and hypoxia by preventing protein aggregation but have established roles in resistance/stress/virulence/dormancy [47]. Rv2031c is regulated by the two-component regulatory system (DosR/DevR regulon) and is a latency stage disease marker [48–49]. Heat shock proteins assist in *M*. *tuberculosis* survival and also provide signal to the immune response [50]. Rv2031c and Rv2005c were predicted as a strong vaccine candidate by Zvi A et al [51].

Rv2462c (trigger factor), involved in protein export, acts as chaperone by maintaining the newly synthesized protein in an open conformation. It was reported to have increased intensity during nutrient deprivation in *M*. *tuberculosis* [52]. Rifat et al [53] reported that its intensity is regulated by inorganic phosphate limitation and suggested to play an important role in the survival of *M*. *tuberculosis* during chronic infection.

Rv2744c (35-kDa antigen), a hypothetical protein, is homologous to phage shock protein A (PspA) of *E*. *coli* and a predominant binding partner and substrate of PepD for proteolysis [54]. It was found to exhibit increased intensity upon exposure to vancomycin and cell wall damaging antibiotics suggesting their role in resistance to cell envelope stress [55]. PepD proteolytically regulates Rv2744c levels to maintain cell wall/cell envelope homeostasis in *M*. *tuberculosis*. It is also speculated that cleavage of Rv2744c by PepD may represent a mechanism for terminating the membrane stress response following cessation of the inducing stimulus. Future studies are aimed at delineating the specific mechanism by which it participates in cell wall homeostasis, and defining the other factors that participate in this stress response pathway.

Rv1479 (MoxR1) is a probable transcriptional regulator involved in regulatory function. Jungblut et al [56] found that four MoxR protein species were with different mobility. Hu et al [57] reported that MoxR1 m-RNA expression was more than four-fold in persisters compared to stationary phase of mycobacteria. Recently Rv1479 has been reported in *M*. *tuberculosis* pellicles [58]. Our study assumes that increased intensity of this protein overcomes the burden of the transcriptional regulation.

Rv0685 (Elongation factor–Tu) is a conserved protein involved in the elongation phase of translation and post-translational modifications. It also has RNA chaperone activities, ensuring that tmRNA adopts an optimal conformation during aminoacylation [59]. Ef-Tu phosphorylation is implicated in acclimation to the stress conditions encountered during the course of infection. *M*. *tuberculosis* phosphoproteome revealed Ef-Tu to be phosphorylated, Sajid et al [60] reported that phosphorylation of Ef-Tu by Protein Kinase B reduced its interaction with GTP, suggesting reduction in protein synthesis. In our study intensity of Rv0685 might be increased due to interruption of translational steps (primary target sites of aminoglycosides) and accumulation of Rv0685 might occur.

In the present study we observed that on performing docking analysis, both drugs interacted with conserved residues of Usp, SDR, PspA and ferritin domain of Rv2005c, Rv0148, Rv2744c and Rv3841 respectively which might alter their functions. It is predicted that these proteins might be exhibiting increased intensities to compensate the effect of drugs. Further we also found pupylation sites in Rv0148, Rv2005c and Rv2744c. Pupylation is a PTM through which small disordered protein Pup is conjugated to lysine residues of proteins marking them for proteasomal degradation. As modification with pup is reversible, pupylation is also likely to have a regulatory role [42]. Pup-proteasome system controlled by pupylation contributes to the virulence/survival strategy of *M*. *tuberculosis* in the host and makes the bacteria more resistant to various stresses [61]. Therefore it is assumed that pupylation of modulated protein (drug-protein complex) might be undergo turnover by proteasome machinery to overcome stress through protein-protein interaction. We assume that increased intensities of proteins might be contributing in imparting resistance against AK and KM. Further, detailed study in this direction may help in searching for new targets for drug development.

## Conclusion

In a nutshell, this is the first report on the membrane and membrane associated proteins of AK and KM resistance in *M*. *tuberculosis* using proteomic coupled with bioinformatic approaches. Among the twelve proteins which were found to have increased intensities only nine were with defined roles and three with unknown functions. Molecular docking showed proper interaction of both drugs with hypothetical proteins (Rv2005c, Rv2744c and Rv0148) as well as ferritin. GPS-PUP analysis suggested presence of pupylation sites within these proteins. It is depicted that increased intensities of these proteins and proteasome sub unit alpha might not only be neutralizing/modulating the drug molecules but are also involved in protein turnover to overcome AK and KM resistance. Apart from that we found three proteins–ferritin, bacterioferritin and elongation factor-Tu, involved in iron storage, homeostasis, detoxification, and regulation/metabolism. We assume that iron regulation/metabolism might be playing some crucial role in contributing resistance to AK and KM. Increased elongation factor-Tu (Rv0685) might be due to interruption of translational steps by these drugs. These findings need further exploitation for the development of newer therapeutic agents or molecular markers which can directly be targeted to a gene/protein responsible for resistance so that an extreme condition like XDR-TB can be prevented, which could ultimately lead to broaden the narrow gauge of new or existing therapeutics.

## Supporting Information

S1 Text MS and MS/MS -. (RAR)

S2 Text MS and MS/MS -. (RAR)

## Abbreviations

TB: Tuberculosis
AK: Amikacin
KM: Kanamycin
2DE: Two Dimensional Gel Electrophoresis
